# The Deciphering of Growth-Dependent Strategies for Quorum-Sensing Networks in *Pseudomonas aeruginosa*

**DOI:** 10.3390/microorganisms11092329

**Published:** 2023-09-15

**Authors:** Tereza Juříková, Hynek Mácha, Vanda Lupjanová, Tomáš Pluháček, Helena Marešová, Barbora Papoušková, Dominika Luptáková, Rutuja H. Patil, Oldřich Benada, Michal Grulich, Andrea Palyzová

**Affiliations:** 1 Institute of Microbiology of the Czech Academy of Sciences, Videnska 1083, 142 20 Prague, Czech Republic; tereza.jurikova@biomed.cas.cz (T.J.); hynek.macha@biomed.cas.cz (H.M.); v.lupjanova@gmail.com (V.L.); maresova@biomed.cas.cz (H.M.); dominika.luptakova@biomed.cas.cz (D.L.); rutuja.patil@biomed.cas.cz (R.H.P.); benada@biomed.cas.cz (O.B.); grulich@biomed.cas.cz (M.G.); 2 Department of Analytical Chemistry, Faculty of Science, Palacky University, 17. Listopadu 12, 771 46 Olomouc, Czech Republic; tomas.pluhacek@upol.cz (T.P.); barbora.papouskova@upol.cz (B.P.)

**Keywords:** *Aspergillus fumigatus*, biofilm, microbial interaction, metabolomic analysis, planktonic cell, *Pseudomonas aeruginosa*, QS system

## Abstract

*Pseudomonas aeruginosa* is recognized as a significant cause of morbidity and mortality among nosocomial pathogens. In respiratory infections, *P. aeruginosa* acts not only as a single player but also collaborates with the opportunistic fungal pathogen *Aspergillus fumigatus*. This study introduced a QS molecule portfolio as a potential new biomarker that affects the secretion of virulence factors and biofilm formation. The quantitative levels of QS molecules, including 3-o-C12-HSL, 3-o-C8-HSL, C4-HSL, C6-HSL, HHQ, PQS, and PYO, measured using mass spectrometry in a monoculture, indicated metabolic changes during the transition from planktonic to sessile cells. In the co-cultures with *A. fumigatus*, the profile of abundant QS molecules was reduced to 3-o-C12-HSL, C4-HSL, PQS, and PYO. A decrease in C4-HSL by 50% to 170.6 ± 11.8 ng/mL and an increase 3-o-C12-HSL by 30% up to 784.4 ± 0.6 ng/mL were detected at the stage of the coverage of the hyphae with bacteria. Using scanning electron microscopy, we showed the morphological stages of the *P. aeruginosa* biofilm, such as cell aggregates, maturated biofilm, and cell dispersion. qPCR quantification of the genome equivalents of both microorganisms suggested that they exhibited an interplay strategy rather than antagonism. This is the first study demonstrating the quantitative growth-dependent appearance of QS molecule secretion in a monoculture of *P. aeruginosa* and a co-culture with *A. fumigatus.*

## 1. Introduction

Most bacteria use growth strategies, starting from planktonic growth through swarming to biofilm formation, which facilitates their living in particular environmental niches. This development is described as a sequential and highly regulated process, with each stage corresponding to a unique profile of extracellular metabolites [1]. For instance, pathogenic bacteria undergo a series of growth phases, facilitating their survival in the host environment. These phases, with distinct cellular phenotypes, also define the extent of the pathogen’s virulence. However, the production of specific metabolites and their subsequent secretion into the surrounding environment may depend on the host-induced defense mechanism. A relationship between the bacterial phenotype and pathogenicity has been demonstrated [2,3], with the typical examples of free-floating bacteria and sessile bacteria forming a biofilm representing a critical stage in the development of infection in up to 80% of human bacterial diseases [4].

The ongoing five-step model of bacterial biofilm development, based on an opportunistic pathogen such as *Pseudomonas aeruginosa*, includes the following phases: reversible and irreversible attachment, biofilm maturation stages I and II, and dispersion [5,6]. Recently, Sauer et al. extensively and critically reviewed the five-step model and suggested a more plausible one with three essential steps: aggregation, growth, and disaggregation [7]. The new model defines biofilm development without the specification of any surface and can be thus applied to aggregated bacteria that do not stick to any solid surface. The *P. aeruginosa* biofilm comprises cell aggregates covered with extracellular polymeric substances (EPS), mainly formed by alginate, Psl, and Pel polysaccharides. Other components are extracellular DNA, proteins, and lipids [8].

*P. aeruginosa* is a ubiquitous Gram-negative bacterium with the potential to infect immunocompromised patients [9] in intensive care units who are suffering from several acute and chronic diseases, such as bloodstream infections, surgical site infections, burns, chronic skin wound infections, respiratory diseases such as cystic fibrosis (CF), and urinary tract infections. *Pseudomonas* spp. are resistant to antibiotics and are currently designated as high-priority critical pathogens on the World Health Organization’s watch list [10]. 

*P. aeruginosa* has developed strategies to dominate in polymicrobial infections through the secretion of toxins, siderophores, and inter-kingdom signals [11]. For example, in addition to *P. aeruginosa*, the respiratory tract is most often colonized by an opportunistic pathogenic fungus, *Aspergillus fumigatus* [12]. In CF patients, *A. fumigatus* colonizes the bronchi; this is accompanied by hypersensitization [13,14,15], allergic bronchopulmonary aspergillosis [16], and bronchitis [17]. This complex mutual relationship between pathogens exhibits antagonistic and cooperative aspects [18]. Several in vitro studies have shown an interaction between these two pathogens by using gene expression studies [19] or the production of antagonistic bacterial compounds that inhibit biofilm formation or the germination of the fungal pathogen [20,21,22,23]. Depending on the stress factors, both microorganisms can develop mechanisms to avoid antagonism and switch to a complex interplay [21,23]. 

Interactions between pathogens are based on the most effective strategies by which each participant continuously responds to the invading mechanisms of the enemy. A key mechanism that [24,25] allows bacteria to rapidly adapt to changing circumstances, the modulation of which has been intensively studied, is the communication between individual cells, known as quorum sensing (QS). This system involves a hierarchy of signaling molecules associated with biofilm formation and virulence regulation in pathogenic bacteria [26]. In *P. aeruginosa*, three QS systems, Las, Rhl, and PQS, are known to control cell behavior through the transcriptional regulators LasR, RhlR, and MvfR (PqsR) [27,28]. Together, these QS systems regulate the expression of more than 10% of *P. aeruginosa* genes [29], encoding proteins that synthesize extracellular molecules and regulate virulence gene expression and biofilm development [30]. Various QS-regulated molecules, such as homoserine lactones (HSLs), quinolones, and phenazines, can interact with host cells to influence a wide range of responses, including immunomodulation [31]. Phenazines constitute a large group of bacterial secondary metabolites with a broad range of physiological functions, effects on biofilm formation [32], and roles in the regulation of gene expression [33], including antibacterial [34] and antifungal effects [35]. *P. aeruginosa* produces several phenazines such as pyocyanin (PYO), phenazine-1-carboxamide (PCN), phenazine-1-carboxylic acid (PCA), and 1-hydroxyphenazine (1-HP). They inhibit growth by generating reactive oxygen species and reactive nitrogen species, which cause damage to the mitochondrial ultrastructures of hyphae. PYO and PCN are kept within biofilms due to binding to extracellular DNA (eDNA). PYO also promotes the release of eDNA from the biofilms through cell lysis mediated by H_2_O_2_ [36]. 

*P. aeruginosa* produces two types of HSL molecules, N-3-oxo-dodecanoylhomoserine lactone (3-o-C12-HSL) and N-butanoyl-homoserine lactone (C4-HSL), which bind to the transcriptional activators LasR and RhlR, respectively [37,38], and can lead to the activation of protease production and motility [39]. The third pathway, *pqs*, leads to the synthesis of the quinolone-based molecule 2-heptyl-3-hydroxy-4-quinolone (PQS) and its precursor 4-hydroxy-2-heptylquinoline (HHQ), the signaling of which is mediated by the PqsR receptor [37,38].

Identifying QS molecules as biomarkers is highly desirable due to their high secretion rates, especially for an early stage of infection related to *P. aeruginosa*. This study demonstrated phenotypic variability with multiple structural and metabolic changes in the QS molecule profile, using SEM and MS, respectively, in a monoculture *P. aeruginosa* culture and a co-culture with *A. fumigatus*. Using targeted metabolomic analysis, we showed quantitative changes in the levels of QS molecules in the aggregation phase in a monoculture culture, followed by their dynamics in a co-culture. The obtained results could potentially alter the view on diagnosing and treating nosocomial infections caused not only by *P. aeruginosa* but also by other Gram-negative bacteria that possess the same specific network of QS molecules.

## 2. Materials and Methods

### 2.1. Microorganisms and Growth Media

*P. aeruginosa* PAO1 (ATTC 15692) was purchased from the ATTC collection (Manasses, VA, USA). *A. fumigatus* strain EI278 was isolated from a clinical sample [40]. The media used for the growth of monocultures were Luria–Bertani broth (LB, g/L: 10 tryptone, 5 yeast extract, 10 NaCl, pH 7.0) and a minimal iron-limited medium (M9, g/L: 0.5 NaCl, 3.0 KH_2_PO_4_, 14.62 Na_2_HPO_4_ × 12 H_2_O, and 1.0 NH_4_Cl, pH 7.0) supplemented with trace elements (M9TE, mg/L: 200 MgSO_4_ × 7 H_2_O, 50 CaCl_2_ × 2 H_2_O, 10 ZnSO_4_ × 7 H_2_O, 17 MnSO_4_ × H_2_O, 4.8 CoCl_2_ × 6 H_2_O, 3 CuSO_4_ × 5 H_2_O, 4.5 Na_2_MoO_4_) and with glucose as a source of carbon and energy (M9TEGlu, 10 g/L). For short-term maintenance and conidia harvest, the fungal culture was grown on malt extract agar (MEA, g/L: 45 malt extract, 15 NaCl, 0.44 NH_4_NO_3_, 0.06 MgSO_4_ × 7 H_2_O, 0.0015 CuSO_4_ × 5 H_2_O, 25 agar, pH 5.5) for 5 days at 37 °C. 

### 2.2. Planktonic Culture under Dynamic Conditions

The inoculum for planktonic growth was prepared in two steps. First, 100 mL of the LB medium was inoculated with a vial (1 mL) of a glycerol stock bacterial culture and grown under constant shaking (190 rpm) at 37 °C (inoculum F1). The overnight culture was centrifuged, washed with phosphate-buffered saline (PBS; 0.1 M), resuspended in the M9 medium, inoculated into 100 mL of M9TEGlu medium, and incubated for 12 h at 37 °C (inoculum F2). Fresh suspension of the washed inoculum F2 in PBS (0.1 M, pH 7.0) harvested in the logarithmic growth phase was adjusted to a final CFU/mL (10^5^ CFU/mL). Subsequently, 100 mL of the M9TEGlu medium was inoculated with 1 mL of the fresh resuspended culture of inoculum F2 and grown under constant shaking (190 rpm) at 37 °C. Samples were collected at incubation times of 0, 6, 9, 12, 18, 24, 36, and 48 h to determine the cell viability, metabolic profile, and morphology.

### 2.3. Dual Biofilm Formation in Liquid-Medium Cultures

The co-cultivation of *P. aeruginosa* and *A. fumigatus* was tested in liquid M9TEGlu of the same composition as used for the monocultures (described in Section 2.1). The bacterial inoculum was prepared in two stages using the same procedures as for the monoculture (described in Section 2.2). The fungal strain was incubated on a solid MEA medium (described in Section 2.1) at 37 °C for 5 days. The conidia were harvested by gentle washing with PBS containing 0.01% Tween 80 followed by filtration using a 5 μm syringe strainer (Pluriselect, Leipzig, Germany). The concentration of conidia was measured using a hemocytometer. A fresh suspension of the bacterial culture and harvested conidia from the fungal culture were adjusted to a final concentration of 10^5^ conidia/mL and used as bacterial and fungal inoculum for co-cultivation. The co-cultures were grown on an orbital shaker (190 rpm) at 37 °C for 48 h in three biological replicates. Monocultures of each strain were used as controls. Samples were collected at incubation times of 0, 6, 9, 12, 18, 24, 36, and 48 h to determine the cell viability, metabolic profile, and morphology. To assess the number of viable bacterial cells, serially tenfold diluted samples were spread on the LB agar plates and incubated for 24 h at 37 °C. 

### 2.4. Quantitative PCR Analysis

Quantitative real-time PCR was used for the detection and quantification of fungal and bacterial loads in the co-cultures. DNA was extracted from a freeze-dried homogenate of fungal pellets with adhered bacterial cells (1 mg) from co-cultures. A freeze-dried homogenate of fungal (1 mg) and bacterial (1 mL) monocultures was used as the control. A ZymoBIOMICS D.N.A. Miniprep Kit (Zymo Research, Irvine, CA, USA) was used according to the manufacturer’s protocol with the time extended to 40 min for the continuous mechanical bead beating of the fungal biomass samples with the vortex. DNA was recovered in 100 μL of the elution buffer and stored at −20 °C until analysis. The DNA concentration was determined using a Qubit 2.0 (Invitrogen, Waltham, MA, USA) fluorometer. Quantitative real-time PCR (qPCR) analysis was performed with CFX96 (BioRad, Vienna, Austria) using an iTaq Universal Probes supermix (BioRad) in a total volume of 20 μL with the primers and a probe (Supplementary Table S1), complementary to the ITS1 region of *A. fumigatus* [41] and to the *opr*L gene of *P. aeruginosa* [42] under the cycling parameters outlined in Supplementary Table S1. For the qPCR assay (performed in triplicates), the reaction mixture consisted of 10 μL of 2 × iTaq Supermix, 5 μL template DNA, and each primer (900 nM and 300 nM) for *A. fumigatus* and *P. aeruginosa*, respectively. Dual fluorochrome oligonucleotide hybridization probes with a reporter F.A.M. and a quencher TAMRA were used in a final concentration of 200 nM. Each run included a control sample without template DNA (NTC) and a 6-point standard curve. DNA from *A. fumigatus* (10^8^ conidia/mL) and *P. aeruginosa* (10^9^ CFU/mL) was extracted for absolute quantitation. The theoretical number of genomic copies was calculated using an IDT Inc. calculator from the measured concentration of extracted DNA (ng/μL) and a genome size of approximately 6.2644 Mb for *P. aeruginosa* PAO1 and 28.539 Mb (median total size) for *A. fumigatus* (genome database, NCBI). The standard curves were made using serial tenfold dilution over 10^1^–10^6^ and 10^2^–10^7^ target fungal and bacterial DNAs, respectively. Standard curves (Supplementary Figure S1) and sample quantities were automatically analyzed using Bio-Rad C.F.X. Manager 3.1 software by comparing the samples’ Cq values to the standard curve. Values were expressed as the genome equivalent per mL (calculated from the produced biomass per mL).

### 2.5. Scanning Electron Microscopy (SEM)

All culture samples for the scanning electron microscopy were fixed (4% formaldehyde, 0.1% glutaraldehyde, 0.1 M PBS buffer, pH 7.2) at 4 °C. Then, the fixed samples were centrifuged (9000× *g*, 5 min, RT) and washed with a PBS buffer (0.1 M; 20 min, three times). The bacterial cultures and fungal conidia samples were sedimented onto poly-L-lysine-treated glass coverslips for 24 h at 4 °C. Then, the coverslips were washed with double-distilled water at room temperature and post-fixed with 1% osmium tetroxide for one hour (RT). After the post-fixation process, the coverslips were washed with double-distilled water (20 min, three times) and dehydrated in a graded and absolute ethanol series. Similarly, samples of fungal cultures forming mycelia were post-fixed in 2-mL Eppendorf tubes and dehydrated in a graded ethanol series and absolute ethanol using porous pots (Quorum Technologies Ltd., Ringmer, UK). All the samples were dried in a critical point dryer (K850, Quorum Technologies Ltd., Laughton, UK). Dried coverslips were mounted onto aluminum specimen stubs using wire glue (Anders Products, Andover, MA, USA), and dried fungal mycelia were mounted using ultrasmooth carbon discs (SPI Supplies, Structure Probe, Inc., West Chester, PA, USA). The samples were sputter-coated with 3 nm of platinum in a high-resolution Turbo-Pumped Sputter Coater Q150T (Quorum Technologies Ltd., Ringmer, UK) and examined in an FEI. A Nova NanoSEM 450 scanning electron microscope (Thermo Fisher Scientific, Brno, Czech Republic) was used at 3 or 5 kV using ETD, CBS, and TLD detectors.

### 2.6. Metabolite Extraction

In brief, 50 μL of the culture supernatant was mixed with a 2.5 μL of the internal standard solution (C10-HSL, 1000 ng/mL) and extracted twice with 150 μL of ethylacetate (Honeywell CHROMASOLV™, V.W.R., Stříbrná Skalice, Czech Republic) with 0.1% formic acid (Thermo Fisher Scientific). Ethylacetate extracts were collected in a new microcentrifuge vial and dried using a Savant SpeedVac vacuum concentrator (Thermo Scientific, Waltham, MA, USA) for 2 h at RT. The dried samples were dissolved in 150 μL of 15% acetonitrile (ACN; Honeywell CHROMASOLV™).

### 2.7. Quantitative High-Performance Liquid Chromatography (HPLC) with Tandem Mass Spectrometric (MS/MS) Analysis

HPLC-MS and MS/MS analyses were performed on a Waters Acquity M-class HPLC system (Waters Corporation, Manchester, UK) connected to a Synapt G2-*Si* hybrid Q-TOF mass spectrometer (Waters Corporation) with ESI ionization (2.7 kV) in the positive ion mode. The samples (1 μL) were loaded onto a temperated (40 °C) Hypersil GOLD^TM^ C18 analytical column (3 μm, 1.0 × 100 mm, Thermo Scientific) and separated with a gradient elution as follows: 0–5 min, 5% B; 5–7 min 99% B; 7–10.5 min 99% B; 10.5–11 min 5% B; 11–15 min 5% B, with a flow rate of 50 μL/min. The mobile phases consisted of 0.1% formic acid in water (A) and 0.1% formic acid in ACN (B). 

The full TOF-MS and MSe scans (mass range of *m*/*z* 50–600) were acquired in a high-resolution mode. All analytes were identified using a combination of matches with retention time, accurate mass, and MS/MS pattern. Data were quantitatively processed against an external calibration curve with at least 4 points using Waters MassLynx 4.2 QuanLynx (Waters Corporation, Manchester, UK) software. The LC-MS-MS method was validated in terms of the limit of detection, quantification, linearity, trueness, precision (repeatability), and the reproducibility of the retention time (Supplementary Chapter 1, Table S2). The results are expressed as the arithmetic mean ± standard deviation. The values of one sample, the mixed culture at 12 h, were calculated from two LC-MS measurements.

### 2.8. Relative Quantitation of Pyocyanin via Matrix-Assisted Laser Desorption Ionization MS

The samples were extracted using the liquid–liquid extraction procedure described by Patil et al. [43]. The evaporated residues were reconstituted in 50 μL of 50% LC-MS-grade A.C.N. The samples (0.5 μL) were manually spotted onto a ground steel MTP (Bruker Daltonics, Bremen, Germany) in triplicates and allowed to dry at RT. The α-Cyano-4-hydroxycinnamic acid matrix (10 mg/mL in 50% ACN with 0.1% trifluoroacetic acid) was sprayed over the sports using a SunCollect sprayer (SunChromGmbH, Friedrichsdorf, Germany) with the following parameters: a nitrogen gas pressure of 2.5 bar, 20 horizontal passes, a line distance of 2 mm, the flow rate increasing from 5 μL/min to 25 μL/min, speed in the X/Y direction of 700/1700 mm/min, and a Z position of 35 mm. MALDI MSI analysis was performed using a 12T Solarix FTICR mass spectrometer (Bruker Daltonics, Billerica, MA, USA) equipped with a SmartBeam II 2 kHz laser in a positive mode within a mass range of *m*/*z* 100–500 and the continuous accumulation of the selected ion mode with a 100 Da mass window. The optimized MALDI MSI parameters were as follows: laser energy of 70% with a frequency of 1000 Hz, 50 shots per pixel, a TOF delay of 0.35 ms, and transfer optics of 6 MHz. Data were acquired with a 90 μm lateral resolution. All acquired data were normalized to root mean square and processed in the SCiLS Lab software (v.2023c Pro, Bruker Daltonics, Bremen, Germany). For compounds of interest, the average area under the curve within the sample spot from the triplicates was used for data exploration. 

## 3. Results and Discussion

### 3.1. QS Expression Dynamics in a Monoculture of P. aeruginosa

**Figure 1:**
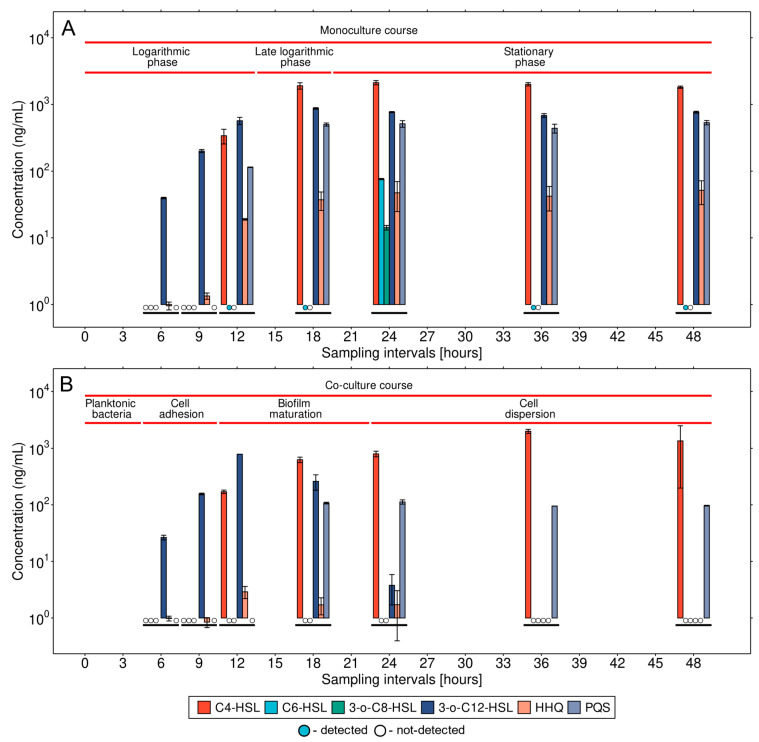
Quantification of extracellular QS molecules secreted during the dynamic growth of a monoculture (**A**) and bacterial biofilm formation of *P. aeruginosa* on the hyphae surface of *A. fumigatus* (**B**). The bar in the graph represents the averages from two biological replicates. Error bars are presented as the standard deviation. 3-o-C12-HSL, N-(3-oxodecanoyl)-homoserine lactone; 3-o-C8-HSL, N-(3-oxo-octanoyl)-homoserine lactone; C4-HSL, N-butyryl-homoserine lactone; C6-HSL, N-hexanoyl-homoserine lactone; HHQ, 4-hydroxy-2-heptylquinoline; PQS, 2-heptyl-3,4-dihydroxyquinoline.

The quantitative MS analysis of the monoculture cell-free supernatants proved the presence of six QS molecules, including 3-o-C12-HSL, 3-o-C8-HSL, C4-HSL, C6-HSL, HHQ, and PQS (Supplementary Figure S2), the molecules that play an essential role in intercellular communication. However, considerable variability occurred in those molecules’ levels during the monoculture growth phases (Figure 1A). The acyl homoserine lactones C6-HSL and 3-o-C8-HSL were detected in significant amounts in the long-term stationary phase, with maximum levels of 76.0 ± 1.6 ng/mL and 14.2 ± 1.1 ng/mL, respectively, when the aggregated cells embedded in EPSs. This correlates well with the previous study [44] depicting both the C6-HSL and 3-o-C8-HSL as less abundant QS signal molecules in *P. aeruginosa*. The level of the other acyl homoserine lactones, differing in the acyl chain length or oxidation state at the acyl C-3 position (C4-HSL and 3-o-C12-HSL), varied depending on the individual growth phases. The 3-o-C12-HSL molecule was detected in all growth phases of the monoculture. The highest level reached a maximum of 872.2 ± 24.7 ng/mL in the late logarithmic phase. The 3-o-C12-HSL concentration also remained high at bacterial aggregation and in the stationary phase when the bacteria already constituted a biofilm. *P. aeruginosa* is known for its disposing of hierarchically arranged QS systems, with the *las* system being superior to all others. This results in the preferential synthesis of 3-o-C12-HSL via the *lasI*/*lasR* quorum-sensing system [45]. Moreover, previous studies have shown that the concentration of the 3-o-C12-HSL molecule can vary significantly depending on the growth conditions and culture type [46,47]. In contrast to 3-o-C12-HSL, the C4-HSL first appeared at the end of the logarithmic phase and persisted at increased levels in the late growth phases. In aggregated cells, the C4-HSL achieved a three-fold higher concentration of 2122.9 ± 155.2 ng/mL (Figure 1A) compared with its concentration at the end of the logarithmic phase. As a result of its superiority, LasI directs the synthesis of C4-HSL by controlling the *rhl* QS system. Moreover, the beneficial role of 3-o-C12-HSL constitutes its ability to regulate the expression of several genes involved in activating the subordinate *rhl* system [48], where C4-HSL is the most significant autoinducing molecule. The high level of C4-HSL expression is in agreement with the fact that the C4-HSL supports a significant function in biofilm development [49]. The QS system uses acylated homoserine lactones as cell density markers. The activation of the *rhl* system by C4-HSL is often associated with stress conditions such as nutrient limitation in the late stages of growth [50]. 

The complex system of QS molecules includes phenazines as uniquely pigmented metabolites involved in virulence and competitive fitness, which have potential as biomarkers in infectious diseases [34]. In a recently published clinical study using signal molecules based on quinolones as biomarkers, the detected concentrations clearly correlated with live bacterial load of *P. aeruginosa* in sputum, plasma, and urine measured by qPCR [51]. Another component of the QS system is an alkylquinolone, 2-heptyl-4-quinolone (HHQ). Its presence was already detected during the logarithmic phase of monoculture growth, with a stable level of 51.6 ± 20.1 ng/mL until 48 h of monoculture (Figure 1A). Thus, HHQ could significantly contribute to intercellular communication and biofilm development at the level of the aggregation of cells not attached to the surface. MS proved the presence of HHQ in the cell-free medium during the early and mid-logarithmic phases at low but analytically valid concentrations. LasR positively affects PqsR, the transcriptional regulator of the *pqsABCD* operon, as well as controlling the biosynthesis of HHQ. [28,51]. The potential of HHQ as a diagnostic biomarker for pathogenic *P. aeruginosa* was confirmed by its detection in breath condensate and urine samples from critically ill patients [52]. The quinolone-based QS system acts mainly through 2-heptyl-3-hydroxy-4-quinolone (PQS). Its maximum level was 10 times higher than HHQ (533.5 ± 40.1 ng/mL), with consistently stable levels during the late growth stages (Figure 1A). The explanation for this finding can again be found in the superiority of the *lasR* QS system. The activated LasR regulator not only activates the biosynthesis of HHQ, but also affects the expression of *pqsH*. This gene encodes the PqsH enzyme of the biosynthesis pathway, converting HHQ to PQS [28]. This correlates with HHQ and PQS timeline detection. HHQ was produced in the early growth phases, starting from six hours of cultivation, while PQS production was observed after twelve hours of cultivation. The importance of PQS has been proven even in other biological systems (e.g., lipopolysaccharide biosynthesis, type 6 secretion machinery) that use quinolone signaling and play a key role in the pathogenicity and adaptability of the microorganisms in the host [53]. 

### 3.2. Detailed Analysis of QS Molecules in the Co-Culture

**Figure 2:**
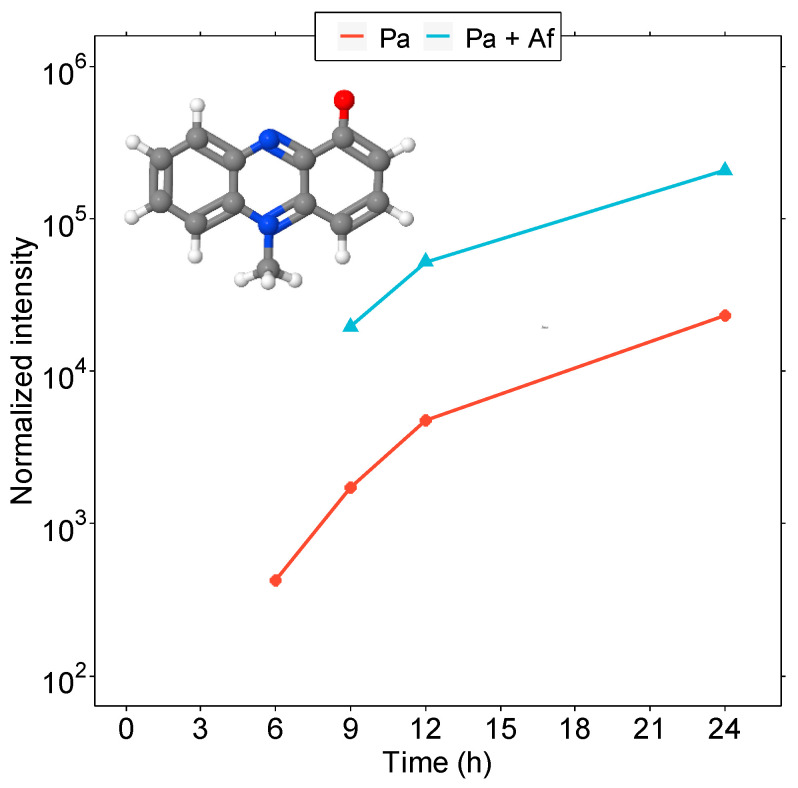
The secretory levels of pyocyanin in the bacterial monoculture (red line) and its interplay with *A. fumigatus* (blue line). Pyocyanine graphic: PubChem CID 6817. Atoms are represented by spheres of different colors (black represents carbon, white represents hydrogen, red represents oxygen and blue represents nitrogen).

The situation was completely different when we compared the metabolic QS profile of the *P. aeruginosa* monoculture and the profile of the bacterium in interaction with *A. fumigatus*. The first significant differences could be observed during the growth stage of the co-cultures when the bacteria predominantly occurred in the planktonic phase and started to attach to the fungal hyphae. No PQS was detected in this case, while the presence of the PYO toxin was confirmed, and its production continued to increase over time (Figure 2). We assume that PYO, one of the key antifungal molecules, was preferentially produced over PQS in the presence of the fungus. Its production is regulated in response to the Las and Rhl homoserine lactone signal molecules and newly found protein QscR, a LuxR-type member, playing an important role in pyocyanin synthesis in *P. aeruginosa* [54,55]. Although the onset of PYO secretion was delayed by three hours, its abundance reached higher values in the co-culture (Figure 2). However, SEM observations of hyphal morphology showed no remarkable changes (see below. The inhibitory effect of PYO on the growth of the fungal mycelium, its germination, and hyphal branching depends on several stress factors, such as the iron concentration, pH, and the concentration of the toxin itself [35,56]. Our studies were performed in iron-limiting conditions to maintain stress conditions. A possible basis of cooperation between the species is the fact that phenazine metabolites drive positive feedback, which enhances bacterial biofilm formation and facilitates the establishment and growth of *A. fumigatus* through iron bioavailability [57]. 

The concentration of other QS signaling molecules was not comparable to that of the *P. aeruginosa* monoculture. We observed a significant difference in their levels at the stage when bacteria completely covered the hyphal surface at 12 h of incubation. Only 50% (170.6 ± 11.8 ng/mL) of the C4-HSL concentration was detected, while the 3-o-C12-HSL concentration was about 30% higher (784.4 ± 0.6 ng/mL) than that of the monoculture (Figure 1B). On the other hand, only a very low concentration (2.9 ± 0.7 ng/mL) of HHQ and no PQS was detected. With bacterial biofilm maturation after 12 h of cultivation, the production of the C4-HSL signal molecule increased systematically in the course of dispersing of bacterial cells from the maturated biofilm, reaching the highest concentration value of 1981.5 ± 162.5 ng/mL at 36 h. In the case of the 3-o-C12-HSL molecule, despite the high concentration at 12 h, the production decreased with biofilm maturation during the following 12 h and was stopped entirely at 36 h (Figure 1B). On the contrary, PQS first appeared at 18 h of co-culture and remained at a similar level up to the end of the culture incubation. The concentration reached lower values, with a maximum value of 112.7 ± 9.8 ng/mL at 24 h, compared to the monoculture (512.9 ± 57.9 ng/mL). The lower concentration of PQS and the absence of the 3-o-C12-HSL molecule could affect C4-HSL production and the overall expression of the *rhl* QS system [28,58]. As a result, a lower concentration of C4-HSL was probably observed compared to the monoculture. The HHQ expression was observed in planktonic cells and in the course of biofilm maturation; however, its production reached a maximum value of only 2.9 ± 0.7 ng/mL at 12 h, compared with the *P. aeruginosa* monoculture (18.9 ± 0.5 mg/mL and maximum value of 51.6 ± 20.1 mg/mL at 48 h). 

### 3.3. Microbial Interaction in the Co-Culture

We monitored the presence of floating bacteria in the co-culture via colony counting (Supplementary Figure S3C). However, the curve course substantially differed from the growth curve of the monoculture. The concentration of viable cells of *P. aeruginosa* in the medium reached the maximum of 1.0 ± 0.4 × 10^8^ CFU/mL at 12 h, which was four times lower than the monoculture (4.0 ± 0.6 × 10^8^ CFU/mL) (Supplementary Figure S3). During this period, the bacteria covered almost all the hyphal surfaces (see below). Then, at 24 h, we detected an extreme decline in planktonic cells (1.0 ± 0.5 × 10^6^ CFU/mL) in the co-culture compared to the monoculture (3.0 ± 0.8 × 10^9^ CFU/mL). Based on the above-given data, conventional microbiological techniques cannot follow the co-culture dynamics in the complex. Therefore, for the quantification of the microorganisms in the co-culture, the qPCR method was used, which provided a detailed genomic load that revealed the total number of bacteria in the system. 

Cell dispersion was evident at 48 h of incubation of the co-culture, when the concentration of viable cells in the medium reached approximately one order of magnitude higher (5.0 ± 0.7 × 10^7^ CFU/mL) than the previous culture duration (24 h) (Supplementary Figure S3). Detachment is an active process that allows for the colonization of new niches and can be caused by several factors, such as external influences, intracellular processes, and the release of EPSs or cell-surface-binding proteins [5]. In addition, the total fungal biomass in the co-culture was weakly reduced to 257.2 ± 18.0 mg compared to the 319.5 ± 16.9 mg of the monoculture biomass after 48 h of incubation (Supplementary Table S3). Multiple factors, such as nutrients, pH, agitation, aeration, the inoculum level, the substrate concentration, polymer additives, the viscosity of the medium, and surface-active agents, are involved in influencing fungal pellet formation in submerged cultures [59,60,61,62,63]. The change in pH values during co-culture could lead to the formation of small-sized pellet forms and a reduction in the total biomass yield, which was observed in the present study (Supplementary Table S3).

**Figure 3:**
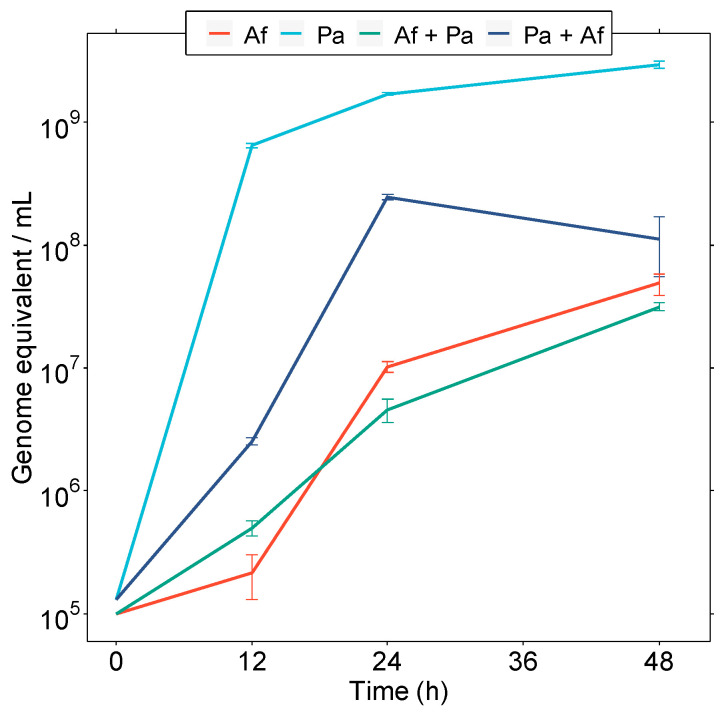
Quantification via qPCR of microbial biomass in co-cultures. The *A. fumigatus* culture alone (red line) and in co-culture with *P. aeruginosa* biofilm (green line), or the *P. aeruginosa* culture alone (light blue line) and in a co-cultured biofilm with *A. fumigatus* (dark blue line).

Due to the bacterial adhesion to the fungal hyphae in the co-culture, conventional microbiology methods such as CFU caused some difficulties (Supplementary Figure S3). Therefore, we used species-specific qPCR to quantify the biofilm-forming bacteria sticking to the fungal hyphae. This showed that, in the co-culture, *P. aeruginosa* culminated at 24 h when the biofilm reached 2.5 ± 0.1 × 10^8^ genome equivalent/mL (Figure 3). The same approach showed that *A. fumigatus*’s biomass increased over time, with the highest increase between 12 and 48 h, culminating in a maximum of 3.1 ± 0.2 × 10^7^ genome equivalent/mL. The number of genome equivalents of *A. fumigatus* in the monoculture was within the same order of magnitude (4.8 ± 0.9 × 10^7^) (Figure 3). All these results suggest that the presence of *P. aeruginosa* in the co-culture did not affect the growth of *A. fumigatus*, while most research studies describe their interaction as antagonistic [18,20,21]. 

### 3.4. Morphological Characterization of the P. aeruginosa Monoculture and Biofilm Formation in the Presence of A. fumigatus

**Figure 4:**
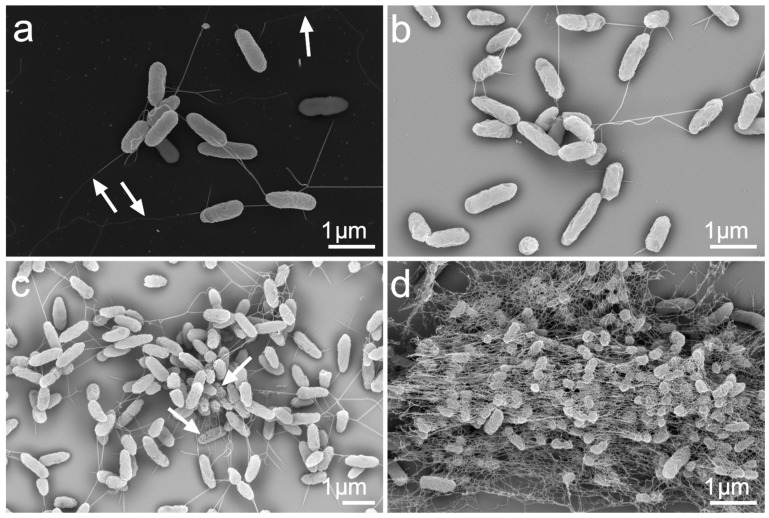
*P. aeruginosa* growth stages in the monoculture over the course of 48 h. SEM imaging in secondary (**a**) and backscattered (**b**–**d**) electrons. (**a**) 12 h—motile bacteria with flagella (arrows). (**b**) 18 h—the bacteria transition to the stationary phase; visible cell wall shrinking. (**c**) 24 h—aggregates with fibrillar connections; some cells have perforations of the cell wall (arrows). (**d**) 48 h—the late stationary phase; massive cell aggregates in extracellular polymeric substances with a dense fiber network. Primary magnification of SEM images: 50,000× (**a**,**b**,**d**), 35,000× (**c**).

In the *P. aeruginosa* monocultures, SEM analysis was used to assess the detailed bacterial cell morphology. No notable differences in the cells’ morphology were observed during the initial logarithmic growth phase, up to six hours of culturing. The observations confirmed the existence of a homogenous population of individual planktonic cells in a shaking flask. The first aggregates of cells interconnected through dense fibrillar surface structures but not attached to the level of the culturing medium or flask walls appeared at 24 and up to 48 h after inoculation (Figure 4). These filamentous structures could be type IV pili, which are known to promote intercellular association in *Pseudomonas* [64], or pseudopilli, as described by Durand et al. [65]. However, two or three bacterial cells in tight contact appeared at the 12 h growth stage. Some possessed flagella, indicating their persisting motility (Figure 4a). The 18 h culture transitioned from the late logarithmic phase to the stationary phase (Figure 4b). The bacteria had already lost the flagella, and their surfaces showed signs of shrinkage, although some cells were still passing through cell division. At 24 h, we observed larger aggregates of interconnected cells with diverse morphology. Some cells were in good shape, while others were shrunken and had perforated cell walls (Figure 4c). Finally, after 48 h, cells with a wrinkled surface were glued together in EPSs, exhibiting a dense fiber network (Figure 4d). 

We used SEM to follow the development and time-associated changes in the morphology of fungal mycelia and bacterial cells in the bacteria–fungus co-cultures at pre-defined growth points. Because the fungal hyphae polarized growth and due to the shear forces occurring in the shaken cultures [66], typical pellet formation appeared in both the monoculture and the co-cultures after the ninth hour. The observed pellets’ morphology suggested their formation via a coagulative process [67]. 

**Figure 5:**
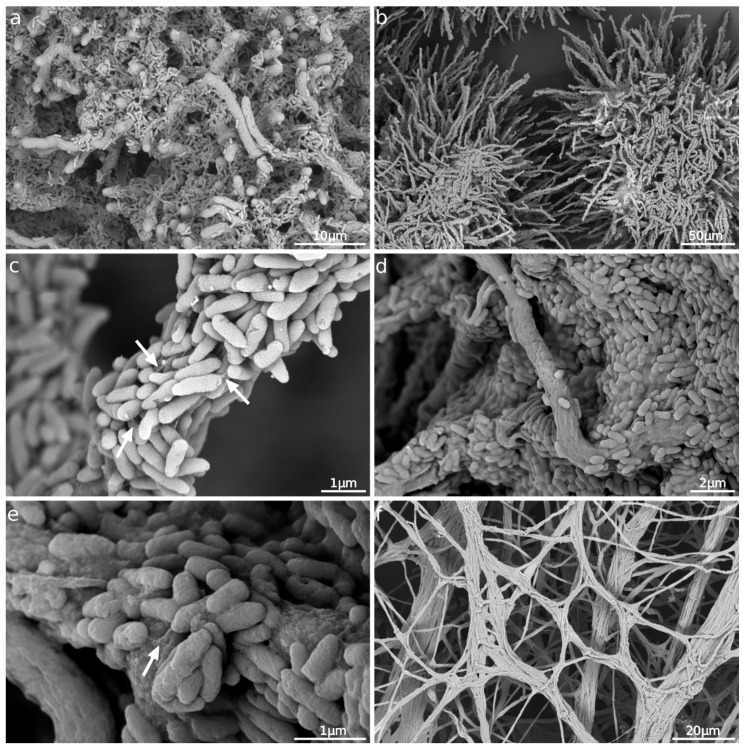
SEM imaging in secondary electrons; co-cultures of *P. aeruginosa* and *A. fumigatus*. (**a**) 9 h—bacteria covering fungal hyphae. (**b**) 12 h—fungal pellets with bacteria covering almost all accessible hyphal surfaces. (**c**) 12 h—detail of bacteria connected by fibrillar structures (arrows). (**d**) 18 h—bacteria embedded in extracellular polymeric substances. (**e**) 18 h—detail of bacteria with pili (arrow) in extracellular polymeric substances. (**f**) 48 h—detail of fungal pellet without bacteria. Primary magnification of SEM images: 8000× (**a**), 1200× (**b**), 50,000× (**c**), 25,000× (**d**), 80,000× (**e**), 3500× (**f**).

In the co-cultures, after the ninth hour, the bacteria started attaching to the fungal hyphae on the pellets’ surfaces (Figure 5a). After 12 h of co-culturing, bacteria covered almost all the accessible hyphal surfaces (Figure 5b), with some fibrillar structures connecting them (Figure 5c). Nevertheless, the floating bacterial cells or their aggregates remained in the medium. After 18 h, the bacterial biofilm formed on the pellets exhibited a typical biofilm structure consisting of extracellular polymeric substances (Figure 5d) and occasional filaments representing flagella (Figure 5e). All the observed bacteria had firmly stuck to the fungal hyphae, as the washing steps did not remove them during the process of preparing the samples for SEM. Similarly to the pellet formation, the fungus’s EPSs probably facilitated the observed tight bacteria–hyphae interaction, since it is essential for bacterial attachment [57,66]. 

Compared to the individual growth in the monoculture (Supplementary Figure S4), neither microorganism showed morphologically noticeable differences, indicating their co-existence rather than their competition. The fitness of both microorganisms became worse after 24 h. Characteristic fungal cell wall perforation and the flattening of the hyphae observed in the co-culture were the same in the fungal monoculture, indicating the natural symptoms of the aging fungal organism. A partial detachment of the bacterial biofilm from the fungal pellets started simultaneously. Significantly fewer bacteria stayed bound to the fungal hyphae after 36 h. 

Finally, no bacterial biomass remained associated with the hyphae after 48 h (Figure 5f), but living floating bacteria or their aggregates survived in the co-culture. The cause of such a rapid and absolute dissociation of the bacterial biofilm is unknown. One possible explanation concerns the changes in the fungal cell wall, the physiological properties of which are essential for bacterial binding [66]. Additionally, we should not exclude the physiological processes leading to the detachment of the *P. aeruginosa* cells from the biofilm after their entry into the stationary phase [68]. 

## 4. Conclusions

Infectious diseases continue to constitute a significant global problem. This has led to the characterization of new biomarkers that would enable early, rapid, and reliable diagnosis with targeted therapy. *P. aeruginosa* causes a wide range of infections, leading to approximately 559,000 deaths in 2019, and it is considered a life-threatening pathogen [69]. Given this scenario, QS molecules that regulate the secretion of virulence factors and biofilm formation may play an important role in therapeutic strategies. QS systems are responsible for the secretion of signaling molecules into the extracellular environment if the concentration of bacteria rises above a certain concentration threshold. As a result, such compounds have the potential to indicate virulence and thus enable its early identification. This ability to detect signaling molecules as biomarkers in vivo appears promising for the identification of the onset of infection. Perhaps the best known and most urgent case where this approach can be applied is in patients suffering from cystic fibrosis. 

This study focused on quantitatively determining the QS molecule portfolio in *P. aeruginosa* with the potential to act as a new biomarker. The MS analysis indicated that the metabolic changes during the transition from planktonic to sessile cells, with subsequent biofilm formation, are undoubtedly complex. We have shown that, in the biofilm stage, bacteria have a completely different QS strategy than planktonic cells. In monocultures, a broad profile of HSL molecules and phenazines was confirmed for all morphological variants. In addition, we used the interaction with a fungal pathogen as a stress factor. Co-infection with the fungus *A. fumigatus* is often observed in patients suffering from cystic fibrosis, characterized by a worsened course of the disease and the need to indicate a wider spectrum of drugs [18]. The importance of microbial interactions as a consequence of the use of drugs in patients with and without cystic fibrosis has been rigorously investigated by Sass et al. [35]. The interaction is usually mediated via secondary metabolites (pyoverdine, pyocyanin, or pyochelin). In our study, suppression of the bacterial QS network by the fungus in favor of a few dominant QS molecules was demonstrated in these co-cultures. Under these stress conditions, novel findings included the dominant detection and quantification of the HSL molecules, specifically, 3-o-C12-HSL and C4-HSL, produced hierarchically with linearly increasing concentrations in the initial stage of biofilm formation (3-o-C12-HSL), followed by its maturation and cell dispersion (C4-HSL). In addition, a weak detection of HHQ was observed in favor of the PQS molecule. In this context, dispersed cells appear to be morphologically similar to planktonic cells but have a different QS molecule portfolio necessary for their translocation to new colonization sites. Although dispersed cells represent a phase of the biofilm lifecycle that is more vulnerable to antimicrobial agents and immune responses, their active metabolism presents the potential for use in the therapeutic treatment of infections associated with biofilm re-formation. This finding has an important role to play in the case of chronic infections, because pathogens tend to adapt to the colonized organism [18] gradually. The possibility, outlined by us, of quantifying signaling molecules and depicting the signal strength of intercellular communication represents a potential avenue for the detailed monitoring of environmental influences on the co-existence of these two dangerous pathogens. As a result, treatment procedures could be optimized.

Furthermore, it was demonstrated that, despite the production of the PYO toxin, a key antifungal substance, there was an interplay between the two pathogens, without significant bacterial antagonistic effects on the development of the hyphal structure of the fungus. The individual growth of both organisms was quantified using qPCR with the parallel determination of the total biomass dry weight and colony counting methods. This analysis was supported by detailed scanning electron microscopy observations, demonstrating bacterial morphological changes, including the transition from planktonic growth to biofilm formation. Considering the interactions of *P. aeruginosa* and *A. fumigatus*, our data support the co-existence theory rather than a significant competitive relationship. This corresponds with the clinical observation that these pathogens commonly co-colonize the airways of patients with chronic lung disease. 

## Data Availability

Raw data will be provided by the corresponding author upon reasonable request.

## Supplementary Materials

The following are available online at https://www.mdpi.com/article/10.3390/microorganisms11092329/s1, Chapter 1: HPLC-MS/MS method validation. Figure S1: Amplification standard curve of target sequence by QPCR for (A) *A. fumigatus* (ITS region) and (B) *P. aeruginosa* (*opr*L gene) generated using Bio-Rad CFX Manager 3.1 software; Figure S2: Extracted ion chromatograms from standards of C4-HSL ([M+H]+ = 194.0767 *m*/*z*), C6-HSL ([M+H]+ = 222.1111 *m*/*z*), C8-HSL ([M+H]+ = 250.1413 *m*/*z*), and 3-o-C8-HSL ([M+H] = 264.1209 *m*/*z*), 3-o-C12-HSL (([M+H]+ = 320.1834), PCA ([M+H]+ = 225.0669), PCN ([M+H]+ = 224.0795), PQS ([M+H]+ = 260.1644), HHQ ([M+H]+ = 244.1661) in a particular retention times; Figure S3: The time course of growth parameters in a monoculture of *P. aeruginosa* and a co-culture of *P. aeruginosa* with *A. fumigatus*. Figure S4: *A. fumigatus* monoculture, SEM imaging in backscattered electrons. Table S1: Primers, probes, and quantitative PCR (qPCR) cycling parameters. * Probes bear a -5′6-carboxyfluorescein [FAM] reporter dye and a 3′ carboxytetramethylrhodamine [TAMRA] quencher; Table S2: Validation parameters for the HPLC-MS/MS method. Table S3: Comparison of the growth parameter (cell dry weight) of *A. fumigatus* performed in co-culture or monoculture.
