# Supplementary material for: The Deciphering of Growth-Dependent Strategies for Quorum-Sensing Networks in Pseudomonas aeruginosa
Source: Microorganisms. 2023 Sep 15;11(9):2329. doi: 10.3390/microorganisms11092329 (PMC10534576; doi:10.3390/microorganisms11092329)
Supplement: Supplementary file 1 [file microorganisms-11-02329-s001.zip › microorganisms-2554164-supplementary.pdf]

Supplementary file

The Deciphering of Growth-Dependent Strategies of Quorum Sensing Network in *Pseudomonas aeruginosa*

Tereza Juříková [1], Hynek Mácha [1, 2], Vanda Lupjanová [1], Tomáš Pluháček [2], Helena Marešová [1], Barbora Papoušková [2], Dominika Luptáková[1], Rutuja H. Patil [1, 2], Oldřich Benada [1], Michal Grulich [1] and Andrea Palyzová [1, *]

[1] Institute of Microbiology of the Czech Academy of Sciences, Videnska 1083, 142 20 Prague, Czech Republic; tereza.jurikova@biomed.cas.cz (T.J.); hynek.macha@biomed.cas.cz (H.M.); v.lupjanova@gmail.com (V.L.); maresova@biomed.cas.cz (H.M.); dominika.luptakova@biomed.cas.cz (D.L.); rutuja.patil@biomed.cas.cz (R.H.P.), benada@biomed.cas.cz (O.B.); grulich@biomed.cas.cz (M.G.); palyzova@biomed.cas.cz (A.P.)

[2] Department of Analytical Chemistry, Faculty of Science, Palacky University, 17. listopadu 12, 771 46 Olomouc, Czech Republic; hynek.macha@biomed.cas.cz (H.M.), tomas.pluhacek@upol.cz (T.P.); barbora.papouskova@upol.cz (B.P.); rutuja.patil@biomed.cas.cz (R.H.P.)

* Correspondence: palyzova@biomed.cas.cz; Tel.: +420-241062617

	Page
**Chapter 1:** HPLC-MS/MS method validation	2
**Table S1:** Primers, probes and quantitative PCR (qPCR) cycling parameters.	2
**Table S2:** Validation parameters for HPLC-MS/MS method	2
**Table S3**: Comparison of the growth parameter (cell dry weight) of *A. fumigatus* performed in co-culture or monoculture.	3
**Figure S1**: Amplification standard curve of target sequence by QPCR for A) *A. fumigatus* (ITS region) and (B) *P. aeruginosa* (*opr*L gene) generated with Bio-Rad CFX Manager 3.1 software. The log copy number (genome equivalent) detected per reaction was plotted vs. quantification cycle value (Cq).	4
**Figure S2:** Extracted ion chromatograms from standards of C4-HSL ($[M+H]^+$=194.0767 *m/z*), C6-HSL ($[M+H]^+$ =222.1111 *m/z*), C8-HSL ($[M+H]^+$ =250.1413 *m/z)*, and 3oC8-HSL ([M+H] = 264.1209 *m/z)*, 3oC12-HSL (($[M+H]^+$ =320.1834), PCA ($[M+H]^+$ =225.0669), PCN ($[M+H]^+$ =224.0795), PQS ($[M+H]^+$ =260.1644), HHQ ($[M+H]^+$ =244.1661) in a particular retention times.	4
**Figure S3.** The time course of growth parametres in monoculture of *P. aeruginosa* and co-culture of *P. aeruginosa* with *A. fumigatus.*	5
**Figure S4:** *A. fumigatus* monoculture, SEM imaging in backscattered electrons.	6

Abbreviations 6

References 7

Chapter 1: HPLC-MS/MS method validation

The validation of HPLC-MS based method covered the evaluation of limit of detection (LOD), limit of quantification (LOQ), linearity (calibration curve) (US Food and Drug Administration (FDA) guidelines for bioanalytical method validation). The validation was performed using a blank centrifuged growth medium sample. The external matrix match calibration curves were prepared from centrifuged growth medium spiked with commercial standards. Method LOD and LOQ values were defined as the lowest concentrations for which the SDs of the intercept equaled 3.3 and 10, respectively, and represented instrumental values. Method LOD and LOQ were calculated by multiplication of instrumental LOD and LOQ by a dilution factor equaled to 3. Independently prepared quality control samples were run throughout the studies and used to control calibration drift, the reproducibility of the retention times. The calculated validation parameters are summarized in Table S2.

Table S1: Primers, probes and quantitative PCR (qPCR) cycling parameters. *Probes bear a -5′ 6-carboxyfluorescein [FAM] reporter dye and a 3′ carboxytetramethylrhodamine [TAMRA] quencher.

DNA target	Name	Sequence (5′-3′)	Reference	Cycling conditions
**ITS**	A. fum-F	GCCCGCCGTTTCGAC	41	Initial denaturation step at 95 ˚C for 5 min, followed by 45 cycles of denature-tion at 95 ˚C for 5 sec, and 30 sec of annealing/extension at 60 ˚C.
	A. fum-R	CCGTTGTTGAAAGTTTTAACTGATTAC		
	A. fum-P*	CCCGCCGAAGACCCCAACATG		
***opr*L**	oprL-F	AACAGCGGTGCCGTTGAC	42	
	oprL-R	GTCGGAGCTGTCGTACTCGAA		
	oprL-P*	TGAGCGACGAAGCC		

Table S2: Validation parameters for HPLC-MS/MS method

Parameter	Analyte					
	C6-HSL	C8-HSL	3-o-C8-HSL	3-o-C12-HSL	HHQ	PQS
Retention time (min)	4.38	5.54	4.76	6.67	5.78	6.15
LOD (ng/mL)	4.50	1.40	0.06	0.04	0.02	9.90
LOQ (ng/mL)	15.60	4.50	0.21	0.14	0.08	29.90
Linear range (ng/mL)	25–750	5–250	0.5–75	0.5–250	0.1–75	50–750
Calibration equation	y=0.0153x	y=0.0627x	y=0.1028x	y=0.1245x	y=0.7443x +0.1083	y=0.1145x -3.5101
Pearson correlation coefficient, r	0.9991	0.9975	0.9971	0.9952	0.9968	0.9998
Reproducibility of the retention time (RSD) [%]	0.1	0.1	0.2	0.1	0.1	0.1
Truenes (Recovery) [%]	$LL_{50ng/mL}$: 92 $HL_{500ng/mL}$: 83	$LL_{10ng/mL}$: 138 $HL_{250ng/mL}$: 98	$LL_{10ng/mL}$: 102 $HL_{50ng/mL}$: 87	$LL_{10ng/mL}$: 132 $HL_{250ng/mL}$: 96	$LL_{10ng/mL}$: 108 $HL_{50ng/mL}$: 96	$LL_{250ng/mL}$: 127 $HL_{500ng/mL}$: 95
Precission (RSD) [%]	$LL_{50ng/mL}$: 4 $HL_{500ng/mL}$: 3	$LL_{10ng/mL}$: 3 $HL_{250ng/mL}$: 3	$LL_{10ng/mL}$: 1 $HL_{50ng/mL}$: 1	$LL_{10ng/mL}$: 5 $HL_{250ng/mL}$: 2	$LL_{10ng/mL}$: 3 $HL_{50ng/mL}$: 2	$LL_{250ng/mL}$: 2 $HL_{500ng/mL}$: 2

LL – low level, HL – High level

Table S3: Comparison of the growth parameter (cell dry weight) of *A. fumigatus* performed in co-culture or monoculture in the medium.

**Growth parameter**	**Strains**	**Time (h)**		
		**12**	**24**	**48**
**cdw (mg)**	AF	10.0 ± 0.1	46.6 ± 4.2	319.0 ± 25.0
	AF+PA	12.0 ± 0.3	52.1 ± 6.0	259.0 ± 18.0

Figure S1: Amplification standard curve of target sequence by QPCR for A) *A. fumigatus* (ITS region) and (B) *P. aeruginosa* (*opr*L gene) generated with Bio-Rad CFX Manager 3.1 software. The log copy number (genome equivalent) detected per reaction was plotted vs. quantification cycle value (Cq).

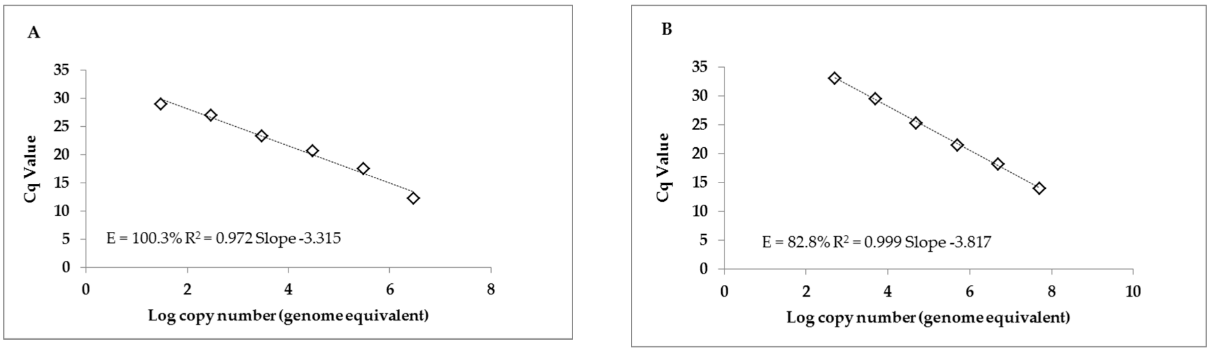

Figure S2: Extracted ion chromatograms and mass spectra from sample supernatant (monoculture) of C4-HSL ($[M+H]^+$=194.0767 *m/z*), C6-HSL ($[M+H]^+$ =222.1111 *m/z*), 3-o-C8-HSL ([M+H] = 264.1209 *m/z)*, 3-o-C12-HSL (($[M+H]^+$ =320.1834), HHQ ($[M+H]^+$ =244.1661), and PQS ($[M+H]^+$ =260.1644) in a particular retention times.

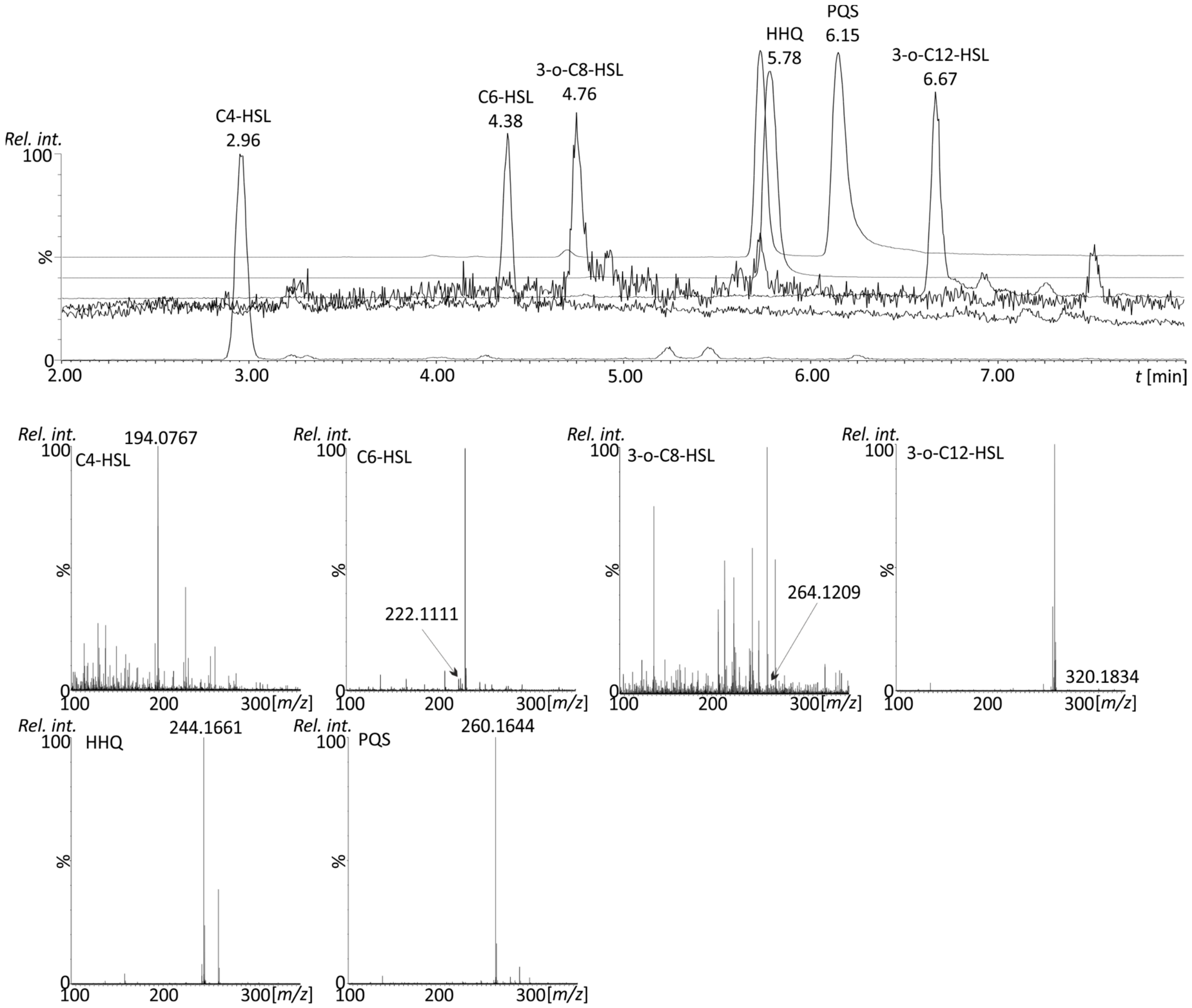

Figure S3: The time course of growth parametres in monoculture of *P. aeruginosa* and co-culture of *P. aeruginosa* with *A. fumigatus.*

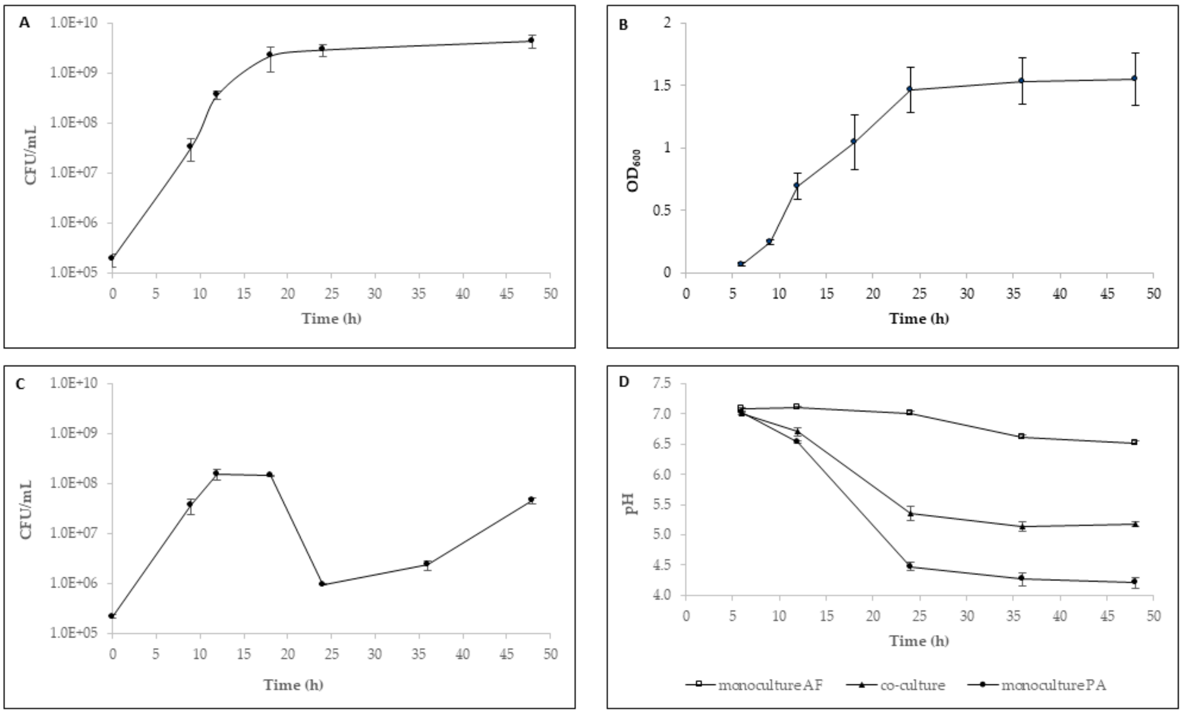

A, B – *P. aeruginosa* monoculture, C – *P. aeruginosa* in co-culture, D – *P. aeuginosa* monoculture, *A. fumigatus* monoculture, co-culture. Data for strain is given as mean ± standard error (calculated from six independent experiments). CFU – colony forming unit, OD_{600} - optical density. AF - *A. fumigatus*. PA - *P. aeruginosa*.

Figure S4: *A. fumigatus* monoculture, SEM imaging in backscattered electrons. (a) 12 h – fungal pellet. (b) 18 h – detail of growing fungal hyphae. (c) 24 h – slight deformations of the fungal hyphae marked by arrows (d) 48 h – fungal hyphae at the end of cultivation with natural symptoms of the fungal aging. Primary magnification of SEM images: 1000× (a), 12000× (b), 6500× (c), 3500× (d).

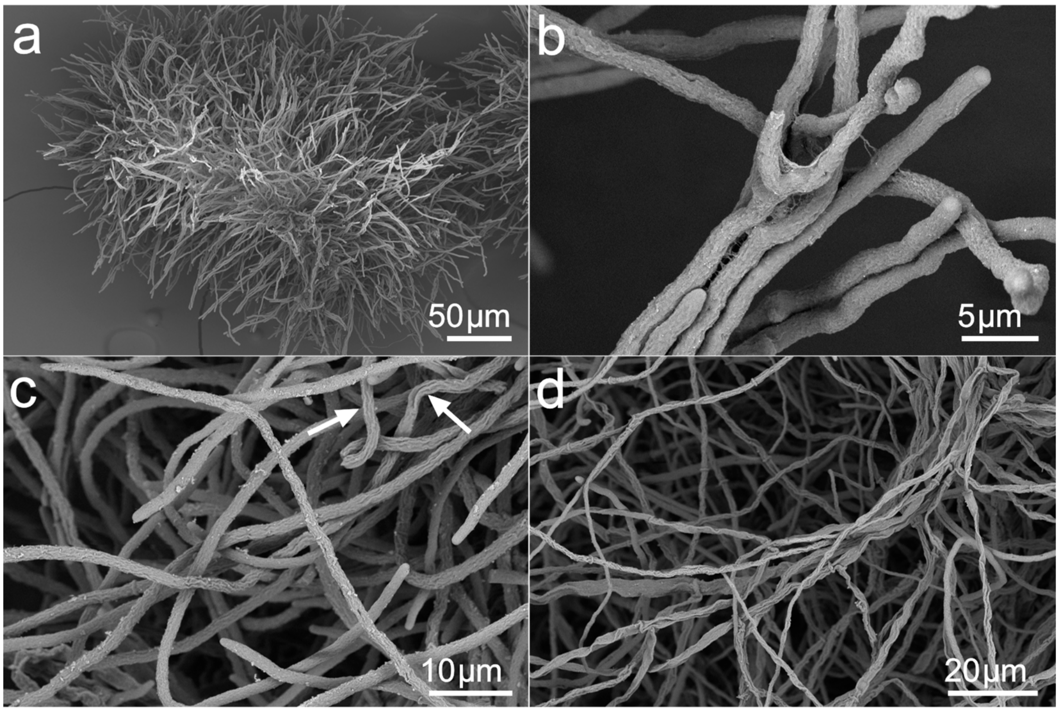

Abbreviations: **AF**, *Aspergillus fumigatus*; **C4-HSL**, N-butyryl-homoserine lactone; **C6-HSL**, N-hexanoyl-homoserine lactone; **C8-HSL**, N-(3-oxo-octanoyl)-homoserine lactone; **CBS**, concentric backscatter detector; **CFU**, colony forming unit; **Cq**, quantification cycle value; **ETD**, Everhart-Thornley Detector; **FAM**, 5′ 6-carboxyfluorescein; **FTICR**, Fourier Transform Ion Cyclotron Resonance; **HHQ**, 4-hydroxy-2-heptylquinoline; **HPLC**, high-performance liquid chromatography; **CHCA**, α-cyano-4-hydroxycinnamic acid; **LB**, Luria-Bertani broth; **LC-MS**, liquid chromatography-mass spectrometry; **LOD**, limit of detection; **LOQ**, limit of quantitation; **M9**, mineral medium; **M9TE**, M9 mineral medium supplemented with trace elements; **MALDI MSI**, matrix-assisted laser desorption/ionization mass spectrometry imaging; **OD** – optical density; **PA**, *Pseudomonas aeruginosa;* **PCA**, phenazine-1-carboxylic acid; **PCN**, phenazine-1-carboxamide; **PQS**, 2-heptyl-3,4-dihydroxyquinoline; **PYO** – pyocyanin; **qPCR**, quantitative real-time polymerase chain reaction; **QS** – quorum sensing; **SEM**, scanning electron microscopy; **TAMRA**, 3′ carboxytetramethylrhodamine; **TLD**, through-the-lens detector; **TOF-MS**, Time-of-flight mass spectrometry; **3-o-C8-HSL**, N-(3-oxooctanoyl)-L-homoserine lactone; **3-o-C12-HSL**, N-(3-oxodecanoyl)-homoserine lactone;

References

41. Walsh, T.J.; Wissel, M.C.; Grantham, K.J. et al. Molecular detection and species-specific identification of medically important *Aspergillus* species by real-time PCR in experimental invasive pulmonary aspergillosis. *J. Clin. Microbiol.* **2011**, *49*, 4150–4157. doi: 10.1128/JCM.00570-11.

42. Joly, B.; Pierre, M.; Auvin, S.; Colin, F.; Gottrand, F.; Guery, B.; Husson, M.O. Relative expression of *Pseudomonas aeruginosa* virulence genes analyzed by a real time RT-PCR method during lung infection in rats. *FEMS Microbiol. Lett.* **2005**, *243*, 271-8. doi: 10.1016/j.femsle.2004.12.012.
